# The statistical foundation of the reference population for semen analysis included in the sixth edition of the WHO manual: a critical reappraisal of the evidence

**DOI:** 10.1093/humrep/deac161

**Published:** 2022-07-18

**Authors:** Alessio Paffoni, Edgardo Somigliana, Luca Boeri, Paola Viganò

**Affiliations:** ASST Lariana, Infertility Unit, Como, Italy; Fondazione IRCCS Ca’ Granda Ospedale Maggiore Policlinico, Infertility Unit, Milan, Italy; Department of Clinical Sciences and Community Health, Università degli Studi di Milano, Milan, Italy; Fondazione IRCCS Ca’ Granda Ospedale Maggiore Policlinico, Department of Urology, Milan, Italy; Fondazione IRCCS Ca’ Granda Ospedale Maggiore Policlinico, Infertility Unit, Milan, Italy

**Keywords:** semen analysis standardization, WHO manual, semen examination, reference values, reference population, World Health Organization

## Abstract

In the most recent version of the ‘*WHO Laboratory Manual For The Examination And Processing Of Human Semen*’, the updated target population used to infer reference values included 3589 fertile subjects, representative of 12 countries and 5 continents, and 10 studies. We have critically evaluated the newly proposed distribution of semen examination results using an approach borrowed from clinical chemistry laboratories and based on the recommendations of the International Federation of Clinical Chemistry for estimation of reference intervals. Surprisingly, most prerequisites to produce common reference intervals through multicentric data were not met. Moreover, when we assessed with the bootstrap method the descriptive reference values obtained from raw data of the 10 individual studies for sperm concentration, sperm number, motility and normal forms, we found that none of the populations was completely correctly described by the reference centiles. We concluded that aggregated data used to build the reference distribution cannot be considered to originate from the same population, and this can result from real differences among individuals or different methodological approaches used in the various studies. Transferability conditions across studies did not seem to have been met. Our findings strengthen the relevance of concerns regarding the use of reference populations in the World Health Organization manual to discriminate between fertile and infertile men.

## Introduction

The ‘*WHO Laboratory Manual For The Examination And Processing Of Human Semen*’ was first released in 1980 from the World Health Organization (WHO) to guide standardization of procedures for the examination of human semen. Since then, five updated versions have been published representing a recognized standard for clinical and research laboratories throughout the world (Wang *et al.*, 2022). Since 2021, the updated sixth edition of the manual has been available as an essential source of the latest evidence-based information in the field of male reproductive health (WHO—World Health Organization, 2021). Although the manual is not intended to be a guideline for clinical decision-making, it is widely used to help clinicians interpret the results of sperm analyses.

The first editions of the manual reported consensus limits to distinguish fertile from infertile subjects. From 2010, the interpretation of the results of the semen examination was modified providing, for comparison, the distribution of values of a reference population consisting of men who have contributed to a natural conception with a time to pregnancy (TTP) of 1 year or less. The 5th percentiles of sperm examination results from those fertile men were proposed as lower reference values (Cooper *et al*, 2010) for normal sperm parameters. Consistent with other laboratory sectors, there was a switch towards the ‘reference values’ instead of the ‘normal values’ (Ozarda, 2016).

Interestingly, in the sixth edition of the WHO manual, the reference population of fertile men has roughly doubled since the 2010 publication and now the updated distribution of target values includes 3589 subjects from 12 countries and 5 continents. The new dataset is freely available and was created after screening the literature with the following inclusion criteria: men with TTP ≤12 months; sexual abstinence range between 2 and 7 days; evidence of compliance with WHO 2010 laboratory techniques; assessment of internal and external quality control (Campbell *et al*., 2021). A total of 10 studies were included (Bonde *et al.*, 1998; Auger *et al.*, 2001; Swan *et al.*, 2003; Haugen *et al.*, 2006; Stewart *et al.*, 2009; Evgeni *et al.*, 2015; Tang *et al.*, 2015; Aboutorabi *et al.*, 2018; Zedan *et al.*, 2018; Lotti *et al.*, 2020). Although this tool represents an important and widespread reference for the assessment of male fertility, its approach has been the object of some criticisms (Esteves *et al*., 2012; Patel *et al.*, 2017).

Some opinion leaders have discussed critically the interpretation of reference values and their relation to fecundity (Boyd, 2010; Skakkebaek *et al.*, 2010; Esteves *et al.*, 2012). Another point of discussion referred to the selection of the population based on TTP ≤12 months. This choice probably encompasses men with different degrees of fertility and, consequently, a shorter TTP was suggested in order to obtain a better reference population for fertile men (Björndahl, 2022). Furthermore, the evaluation of male reproductive function is also made more difficult by the presence of very heterogeneous infertility factors, by the need to deal with female reproductive potential and by the stringent technical requirements needed to ensure standardization (Björndahl and Kirkman Brown, 2022). Not surprisingly, the usefulness of a reference population was questioned, especially because subjects are investigated for overlapping characteristics such as fertility/subfertility/infertility (WHO—World Health Organization, 2021). Following a cautious approach, the latest version of the manual expressly reports a note emphasizing that the lower 5th percentile of a distribution of sperm values does not represent a cut-off limit between fertile and infertile men, and that the centile values are only one way to interpret the results of sperm analysis. This concept has been thoroughly discussed in the context of a ‘paradigmatic shift in the care of male factor infertility’ (Björndahl, 2022).

Although some degree of heterogeneity in the studies used to develop the reference population in the new version of the WHO manual has been recognized, not many details have been provided on how the statistical analysis of the pooled data was carried out. The characteristics of the available dataset do not seem to reflect the common recommendations for developing reference values (Solberg, 2004). Rather, it seems that the data were merged as if they came from a single multicentre study which, however, should respond to greater standardization requirements among the centres involved, such as a common external quality control programme, as well as the control for main confounding factors characteristic of the participants.

Since the interpretation of results from sperm analysis is one of the most relevant topics related to the WHO manual, this manuscript aims to critically evaluate the newly proposed distribution of semen examination results, through analysis of the methodological approach used to define the reference population of the included studies and the findings obtained.

## Methods

The dataset used for 2021 WHO distribution of results is freely available at https://doi.org/10.15132/10000163 and was analysed using the SPSS vers. 20 and Refval 4.11 softwares. Individual studies were evaluated through the compilation of a checklist based on the International Federation of Clinical Chemistry recommendations on estimation of reference intervals (NCCLS EP28-A3 and CLSI and IFCC, 2008) and on the scoring system of BIOCROSS (Wirsching *et al.*, 2018). Since our approach was borrowed from clinical chemistry laboratories and semen analysis is not a biochemical parameter, the checklist was modified from the original recommendations to comply more appropriately with the sperm examination requirements. The checklist was filled in using information reported in the original manuscripts to verify whether the main procedures for the transfer and validation of reference intervals were met.

The Kruskal–Wallis test was used to compare the distributions of parameters among different studies. To review published distributions for quantitative results, our approach was based on European Federation of Clinical Chemistry and Laboratory Medicine (EFLM) recommendations (Henny *et al.*, 2016). From each study included in the WHO manual, raw data were used to describe the distribution of semen analysis results for the reference population of fertile male with a TTP ≤12 months; these private distributions were in turn compared with those of the other studies. This strategy was in line with the EFLM recommendation suggesting that, when a laboratory needs to adopt the reference intervals from an external source (such as WHO manual), it is necessary to verify that the transferability conditions exist and that the critical elements of the original studies are in line both with the operative protocols and the included population. According to EFLM (Henny *et al.*, 2016), an external reference range can be transferred in a subjective way without verification only if several key points are consistent with the working conditions, including geographic and demographic criteria, pre-analytical procedures, analytical performance and statistical methods. Of course, these requirements can be met only under a specific multicentric protocol. On the contrary, the verification of new reference intervals should take place through a specific statistical protocol when the requirements for a subjective validation are not met; to this end, a binomial test can be used to ensure that the number of out-of-range observations is acceptable for the population under study using the specified reference values. If an excess of subjects shows values outside the expected ranges, it will be deduced that the reference values cannot be accepted: the analytical procedure should be revised, or the possible role of confounding factors should be considered in order to elaborate more specific ranges. In fact, the observed differences could be due to the analysis protocols or to real contributions of confounding factors in the population (age, disease, medication, ethnicity etc.) but, in any case, the creation of a single data pool would be questionable.

In the present study, therefore, we wanted to verify how much the distribution of sperm examination results obtained in individual studies included in the WHO 2021 manual is transferable with respect to the other studies. We have considered four main variables (sperm concentration per ml, total sperm count per ejaculate, percentage of total motility, percentage of normal forms) and calculated the reference ranges (5th, 50th and 75th percentiles) with the bootstrap method, excluding in turn each of the 10 studies included in WHO 2021. The bootstrap method consists of repeated random resampling with replacement of the original observations. This is done with appropriate software (Solberg, 2004). After resampling raw data observations 500 times, the percentiles for each study were estimated. Subsequently, the population of each of the studies was compared with the pool of the remaining studies, through the binomial test, to verify the comparability of the results. In this way, for each study, it was possible to obtain information on the acceptability of specific centiles with respect to the values of the WHO manual.

## Results

Although the original studies used to develop the WHO distribution of sperm analysis results described their methods with reference to WHO manual, most prerequisites to produce common reference intervals through multicentric data were not met (Table I). According to the checklist, only a minority of studies were originally designed to elaborate sperm reference intervals, and most of them failed to provide sufficient information on the following important aspects: sample size justification, participation rate, outlier detection/handling, strategies to deal with missing values, blind analysis for laboratory staff, role of ethnicity, variability due to measurements at one point in time. Furthermore, although most of the laboratories adhered to an external quality control programme, this was not systematically stated in all the different studies.

**Table I:** Checklist of prerequisites required to produce common reference intervals through multicentric data for the 10 studies in the World Health Organization manual, 2021.

Items	Aboutorabi * et al.* (2018)	Auger *et al.* (2001)	Bonde * et al.* (1998) ^a^	Evgeni * et al.* (2015)	Haugen * et al.* (2006)	Lotti * et al.* (2020)	Stewart * et al.* (2009) ^b^	Swan * et al.* (2003)	Tang * et al.* (2015)	Zedan * et al.* (2018)
Was the rationale/objectives/hypothesis clearly presented?	Y	Y	Y	Y	Y	Y	Y	Y	Y	Y
Were the inclusion and exclusion criteria for study participation defined?	Y	Y	Y	Y	P	Y	Y	Y	Y	Y
Was a questionnaire designed to reveal inclusion/exclusion/partition criteria in the potential reference individuals?	N	Y	Y	N	N	Y	Y	Y	Y	Y
Was an appropriate written consent form for participation completed?	Y	N	N	Y	Y	Y	Y	Y	Y	Y
Was sample size justification or power description provided?	N	N	N	N	N	Y	N	N	N	Y
Was the participation rate reported with eligible persons at least 50%?	N	N	N	N	N	N	N	N	Y	Y
Were the disease stages or comorbidities of participants evaluated?	N	Y	Y	Y	N	Y	Y	Y	Y	Y
Were the exposures and potential confounders described?	P	Y	Y	Y	N	Y	Y	Y	Y	Y
Did the authors report on methods or outlier detection and handling?	N	N	N	N	N	N	N	Y	N	N
Were any missing values and strategies to deal with missing data reported?	N	N	N	N	N	N	N	Y	N	N
Was the raw effect size estimate (correlation coefficient, beta coefficient) or measure of study precision provided?	Y	P	P	Y	P	P	Y	Y	Y	Y
Were any quality control procedures and results reported (e.g. reported coefficient of variation)?	N	Y	Y	N	N	N	Y	Y	P	Y
Were the analyses blinded for laboratory staff?	N	N	N	N	N	N	Y	N	Y	N
Was standardization of methods properly described with reference to WHO manual?	Y	Y	Y	Y	Y	Y	Y	Y	Y	Y
Was an external quality control programme implemented?	N	N	Y	Y	Y	Y	Y	Y	Y	N
Were reference value data inspected and presented with an histogram or sufficient data to evaluate the distribution?	Y	N	P	Y	P	N	Y	Y	P	Y
Were reference ranges proposed and validated according to established reference values?	N	N	N	Y	Y	P	P	N	P	Y
Was the data discussed in the context of study objectives/hypotheses?	Y	Y	Y	Y	Y	Y	Y	Y	Y	Y
Was the interpretation of the results considering findings from similar studies?	Y	Y	Y	Y	Y	Y	Y	Y	Y	Y
Did the authors acknowledge restricted interpretation due to measurements at one point in time (one single sperm sample)?	N	N	N	Y	N	N	Y	Y	N	Y
Was the possible role of ethnic and racial variability discussed?	N	N	N	N	Y	N	N	Y	Y	Y

Checklist based on Scoring system of BIOCROSS + NCCLS EP28-A3 and CLSI and IFCC (2008); Y, yes; N, no or not reported; P, partially; WHO, World Health Organization.

a In the dataset of raw data referred to as ‘Jensen’.

b In the dataset of raw data referred to as ‘Baker’.

**Figure 1.**
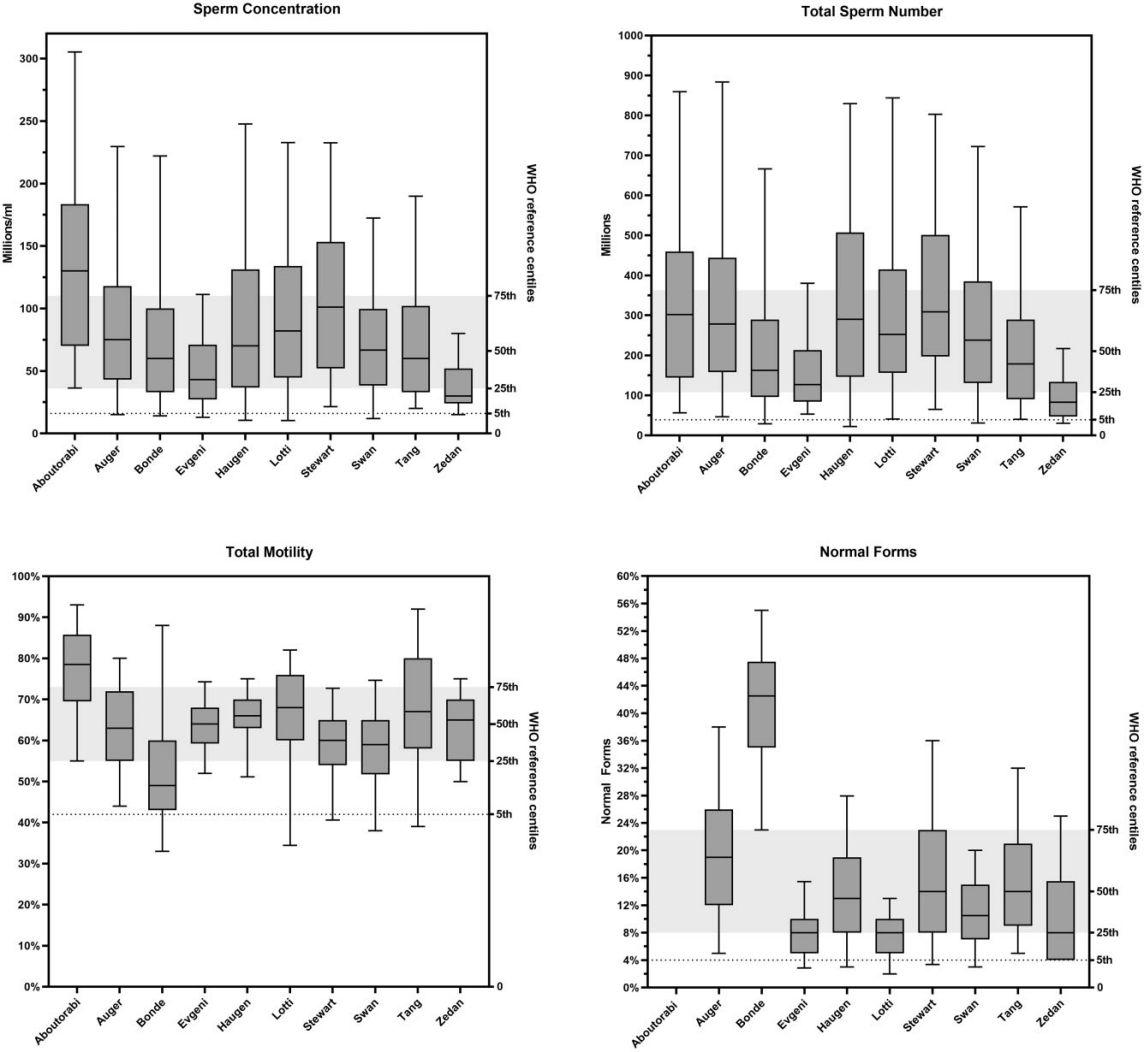
**Sperm concentration, total sperm number, motility and normal forms in 10 different studies.** The descriptive reference values obtained from raw data with the bootstrap method are reported as 5th, 25th and 75th percentiles of the sperm concentration, total sperm number, total motility and normal forms. Boxes represent values between the 25th and 75th percentile; whiskers represent the 5th and 95th centiles. Ranges reported for the reference population in the 2021 World Health Organization (WHO) manual are indicated on the right *Y*-axes: the grey area represents the interquartile range, while the dotted line represents the 5th centile.

The descriptive reference values obtained from raw data with the bootstrap method are reported in Fig. 1 as 5th, 25th and 75th percentiles of the sperm concentration, total sperm number, total motility, and normal forms for each of included studies. As detailed in Table II, the 5th percentile of sperm concentration varied between 11 millions/ml and 36 millions/ml; total sperm number varied between 22 millions and 65 millions sperm/ejaculate; for total motility, the value ranged between 33% and 55% while for normal forms it ranged between 2% and 23%. For comparison, the 5th percentile for the four parameters in the WHO 2021 were as follows: 16 millions/ml, 39 millions sperm/ejaculate, 42% total motility and 4% normal forms.

**Table II:** Descriptive reference values (percentiles) obtained from raw data for main sperm parameters.

	Sperm concentration^c^ (millions per ml)				Sperm total number (millions)				Total motility (%)				Normal forms (%)			
	5th (ref value with 95%CI)^*^	25th	50th (median)	75th	5th (ref value with 95%CI)^*^	25th	50th (median)	75th	5th (ref value with 95%CI)^*^	25th	50th (median)	75th	5th (ref value with 95%CI)^*^	25th	50th (median)	75th
Aboutorabi *et al.*(2018)	36 [27–46]	70	130	183	56 [26–87]	144	302	460	55 [51–59]	70	79	86	\	\	\	\
Auger *et al.*(2001)	15 [12–18]	43	75	118	46 [38–63]	158	279	444	44 [42–45]	55	63	72	5 [4–6]	12	19	26
Bonde *et al.*(1998)^a^	14 [9–17]	33	60	100	29 [16–46]	96	162	290	33 [28–33]	43	49	60	23 [18–28]	35	42	48
Evgeni *et al.*(2015)	13 [11–21]	27	43	71	53 [43–65]	84	127	212	52 [49–55]	60	64	68	3 [2–4]	5	8	10
Haugen *et al.*(2006)	11 [9–19]	37	70	131	22 [5–58]	148	290	507	51 [42–57]	63	66	70	3 [2–4]	8	13	19
Lotti *et al.*(2020)	11 [8–24]	45	82	134	41 [28–93]	157	252	407	34 [28–50]	60	68	76	2 [2–3]	5	8	10
Stewart *et al.*(2009)^b^	21 [18–27]	52	101	153	65 [48–95]	198	308	498	40 [35–45]	54	60	65	3 [3–4]	8	14	23
Swan *et al.*(2003)	13 [10–16]	39	67	100	36 [29–45]	135	240	386	38 [36–42]	52	59	65	3 [2–4]	7	10	15
Tang *et al*. (2015)	20 [18–20]	33	60	102	40 [38–43]	90	178	289	39 [36–43]	58	67	80	5 [4–5]	9	14	21
Zedan *et al.*(2018)	15 [12–15]	24	30	52	30 [22–31]	47	83	133	50 [45–50]	55	65	70	4 [2–4]	4	8	15

* Bootstrap estimates using the non-parametric method. Ref, reference.

a In the dataset of raw data referred to as ‘Jensen’.

b In the dataset of raw data referred to as ‘Baker’.

c Sperm concentration has limited diagnostic power compared to sperm total number according to WHO 2021.

The Kruskal–Wallis test revealed that each of the considered variables (sperm concentration, total sperm number, total motility and normal forms) had a significantly different distribution (*P* < 0.001) for the following comparisons: across 10 studies included in the sixth edition of the WHO manual; across five studies included in the fifth edition of the WHO manual; across five studies newly included in the sixth edition of the WHO manual; between the group of studies included in the fifth version of the WHO manual and that of studies newly included in the sixth edition.

Data reported in each study was compared with the reference values obtained from the pool of the other studies and results are summarized in Table III. None of the populations was completely correctly described by the reference centiles; in fact, in all studies, the percentage of subjects within the 5th, 50th or 75th percentile was significantly higher or lower than expected for at least three out of four parameters (sperm concentration, total sperm number, total motility and/or normal forms). The 5th percentile of total sperm count was the only range that was consistent for most included studies (6 out of 10); however, only two and three studies met the 50th and 75th percentile, respectively. Considering sperm concentration, the 5th, 50th and 75th percentiles correctly classified three, four and one study, respectively. Similarly, considering total motility, the 5th, 50th and 75th percentiles were fully representative for three, two and zero studies out of 10, respectively. Lastly, considering nine studies included for sperm morphology analysis, the three ranges were representative of no more than two studies each. In total, 117 ranges were obtained and compared with those of single studies; in 88 cases (75%), the binomial test revealed that the percentages of samples belonging to the specified ranges was significantly different than expected. In 49 cases (42%), studies showed a higher rate of patients falling in the specified range compared to the 5th, 50th or 75th percentile, revealing an excessive stringency of reference values; conversely, in 39 cases (33%), a lower rate of patients fell in the specified range compared to the 5th, 50th or 75th percentile, corresponding to an excess of subjects showing results above the reference values. As shown in Supplementary Table SI and Supplementary Fig. S1, similar results were obtained considering the following additional variables: sperm volume, progressive motility and vitality.

**Table III:** Comparison of reference values for sperm among different studies.

		Sperm concentration (millions/ml)^c^			Total sperm number (millions/ejaculate)			Total motility (%)			Normal forms (%)		
Study		5th	50th	75th	5th	50th	75th	5th	50th	75th	5th	50th	75th
Aboutorabi *et al.* (2018)	*Reference value*	*15*	*64*	*106*	*33*	*198*	*345*	*41*	*64*	*72*	*4*	*14*	*23*
	Subjects in the range	1%^*^	20%^*^	39%^*^	3%	32%^*^	60%^*^	1%^*^	14%^*^	29%^*^			
Auger *et al.* (2001)	*Reference value*	*17*	*63*	*106*	*39*	*209*	*363*	*40*	*65*	*73*	*4*	*12*	*20*
	Subjects in the range	6%	41%^*^	70%^*^	4%	34%^*^	64%^*^	3%^*^	57%^**^	80%^**^	5%	27%^*^	56%^*^
Bonde *et al.* (1998) ^a^	*Reference value*	*17*	*66*	*110*	*39*	*214*	*368*	*43*	*65*	*73*	*4*	*13*	*20*
	Subjects in the range	9%^**^	56%	81%^**^	7%	65%^**^	83%^**^	25%^**^	80%^**^	89%^**^	1%^*^	1%^*^	4%^*^
Evgeni *et al.* (2015)	*Reference value*	*16*	*67*	*111*	*38*	*212*	*368*	*41*	*64*	*73*	*4*	*14*	*23*
	Subjects in the range	7%	72%^**^	96%^**^	0%^*^	75%	95%	0%^*^	54%	93%^**^	13%^**^	91%^**^	99%^**^
Haugen *et al.* (2006)	*Reference value*	*16*	*66*	*109*	*39*	*208*	*360*	*41*	*64*	*73*	*4*	*14*	*23*
	Subjects in the range	10%^**^	43%	67%	6%	37%^*^	61%^*^	1%	39%^*^	93%^**^	13%^**^	58%	88%^**^
Lotti *et al.* (2020)	*Reference value*	*16*	*66*	*109*	*39*	*209*	*362*	*42*	*64*	*73*	*4*	*14*	*23*
	Subjects in the range	7%	42%	64%^*^	5%	42%	70%	7%	37%^*^	64%^*^	22%^**^	98%^**^	100%^**^
Stewart *et al.* (2009) ^b^	*Reference value*	*15*	*62*	*107*	*38*	*202*	*352*	*42*	*65*	*73*	*4*	*14*	*23*
	Subjects in the range	1%^*^	32%^*^	56%^*^	2%^*^	26%^*^	56%^*^	6%	77%^**^	100%^**^	9%^**^	52%	76%
Swan *et al.* (2003)	*Reference value*	*17*	*66*	*112*	*39*	*203*	*379*	*42*	*65*	*74*	*4*	*15*	*25*
	Subjects in the range	8%^**^	48%	80%^**^	6%	41%^*^	75%	8%^**^	78%^**^	95%^**^	13%^**^	79%^**^	100%^**^
Tang *et al.* (2015)	*Reference value*	*15*	*68*	*114*	*36*	*228*	*397*	*42*	*63*	*70*	*4*	*14*	*24*
	Subjects in the range	3%^*^	55%^**^	81%^**^	4%^*^	63%^**^	86%^**^	6%^**^	37%^*^	58%^*^	5%	53%^**^	80%^**^
Zedan *et al.* (2018)	*Reference value*	*17*	*70*	*113*	*42*	*224*	*380*	*41*	*64*	*73*	*4*	*14*	*23*
	Subjects in the range	9%^**^	90%^**^	100%^**^	20%^**^	97%^**^	100%^**^	2%^*^	48%	93%^**^	28%^**^	75%^**^	92%^**^

Reference values were calculated with the bootstrap non-parametric method excluding the study reported on the left. Subjects in the range are those described in the study reported on the left.

* The percentage of subjects with results in the specified percentile is significantly lower than expected.

** The percentage of subjects with results in the percentile is significantly higher than expected.

a In the dataset of raw data referred to as ‘Jensen’.

b In the dataset of raw data referred to as ‘Baker’.

c Sperm concentration has limited diagnostic power compared to sperm total number according to WHO 2021.

## Discussion

A critical review of the most recent version (sixth) of the WHO manual has recently analysed the major changes in the objectives and methods from the previous edition, also comparing the thresholds of basic sperm parameters (Boitrelle *et al.*, 2021). It was underlined that, even if reference values did not markedly change, the incorporation of additional participants and samples from additional continents provided a higher statistical power to the development of reference ranges. However, here we have shown that methods used for the definition of common distribution values in the sixth edition of the WHO manual are not devoid of methodological weaknesses. Specifically: it is clearly stated that data were not derived from a multicentre study; however, the reference distributions were obtained by pooling together available data from original studies generally not designed to develop reference values and lacking the characteristics needed to be considered multicentre; our statistical analysis excluded that fertile males with TTP ≤12 enrolled in different studies can be considered to originate from the same distribution; the reference values obtainable in individual studies do not match those of the other studies, suggesting that there are significant differences among subjects and/or in the analytical approach. In particular, based on the differences observed among the included studies, it is plausible to think that: the populations considered were different for geographic, ethnic or unexpected variability; different analytical methods were used in the various studies; inter-laboratory variability could not be completely overcome despite efforts to standardize previous versions of WHO manuals. Whether the reason is biological or methodological, or a mixture of the two, the definition of a common distribution of laboratory results does not solve the problem. On the contrary, it tends to exacerbate the shortcoming by highlighting a general lack of comparability and raising doubts on the scientific value of the proposed reference values for fertile men based on this approach.

Support for our observations comes from previous contributions which have elegantly identified key issues related to WHO reference values for semen assessment and the main sources of variability among studies such as the quality of sample examination, the paucity of high-quality data collection from primary studies or the poor geographical spread (Campbell *et al.*, 2021; Vasconcelos *et al.*, 2022).

It is also important to underline that the significant variability herein recognized refers to studies from laboratories with the greatest experience in the world with a certain degree of author overlapping; for this reason, it is legitimate to think that among laboratories with less experience the lack of standardization, and therefore the difficulty in dealing with reference values, is even higher.

The authors of the sixth edition of the WHO manual for human semen analysis provided an important basis for standardization of sperm analysis and firmly stated that the included reference thresholds should not be used as reference values to discriminate between fertile and infertile men because of a degree of inconsistency in the definition of the reference population (Björndahl, 2022). Therefore, the classical simplistic notion of reference values should be abandoned. In particular, the 5th centiles for main semen parameters are deemed useful but insufficient to diagnose infertility, as reported in the WHO manual itself. Our findings further strengthen the relevance of these concerns and shed more light on the inherent limitations deriving from the reference male fertile population.

Some weaknesses of this report should be recognized. First, our methodology is borrowed from clinical chemistry laboratories and therefore could be criticized in its application to the analysis of basic seminal parameters; however, it must be recognized that clinical biochemistry procedures are mostly automated and offer a good level of standardization across laboratories. The semen analysis, characterized by a greater inter-laboratory variability, would require an even higher methodological rigour. Second, our scoring checklists were completed using only published information without contacting the authors for missing data; however, most of the checklist items addressed critical aspects of study protocols, which were generally described, if applicable. For the items considered, missed data were unlikely. Third, while significant differences were evident among the studies, a good agreement could be observed for some values such as the 5th centile of total sperm number and normal forms. Notwithstanding this partial agreement among different studies, the importance of other reference percentiles (such as 50th and 75th) or other parameters (such as total motility) on which the concordance was markedly less, should not be overlooked. Finally, we decided to analyse in depth only four main variables representative of semen quality and not all the semen parameters; however, the inclusion of other variables would have hardly changed the meaning of our findings, as shown in Supplementary Table SI.

Sperm concentration was included in our analysis given its wide use as a measure of sperm quality; however, it is worth mentioning that the WHO manual stated that sperm concentration has limited diagnostic power since it is not a direct measure of testicular sperm production (WHO—World Health Organization, 2021).

Given the limited value of the reference values obtained to date, suggestions to overcome the problem in the future are of utmost importance (Vasconcelos *et al.*, 2022). The great heterogeneity among the studies can be attributed to the specific problems related to the composition and handling of human semen (Björndahl, 2022) or to the plethora of standard operating procedures available, some of which are still not validated for clinical practise, such as the computer-assisted sperm analysis (Tang *et al.*, 2015). In this regard, we believe that efforts to increase the standardization of methods among laboratories and to provide accreditation for basic semen examination are strongly needed. The new version of the WHO manual, together with the ISO standard 23162:2021 (International Organization for Standardization, 2021) for basic semen examination, will be the primary resource. Multicentric studies should be promoted under common external quality controls and protocols, possibly sharing the same samples to improve concordance among laboratories. Likewise, the vital role of an adequate operator training programme and regular internal quality controls in obtaining useful results cannot be underestimated. Under these prerequisites, semen from fertile subjects with a reduced TTP (4 months) enrolled, for example, from couples attending obstetric units for early pregnancy monitoring may be worthy of evaluation. This kind of approach could help us to understand if male fertility can be properly classified according to semen analysis or whether variability exists among different populations according to geographical origin or ethnicity. Alternatively, or pending high-quality data, even if it may seem anachronistic, it could be useful to develop private reference values in different laboratories, based on their own population of fertile subjects and their own methodological approaches. It is worth underlining in this context that semen examination is not only a prognostic tool for fertility treatments but also a proxy of male general and reproductive health. Therefore, a unique reference population may be inadequate to develop useful medical decisions in different conditions (Ventimiglia *et al.*, 2015; Björndahl, 2022; Björndahl and Kirkman Brown, 2022).

In conclusion, we have provided statistical evidence to confirm that the current WHO distribution of semen examination results is not ideal for interpreting male fertility, also owing to the application of insufficiently rigorous methodological approaches. Specific, well designed, large multicentric studies are urgently required to clarify whether the abandonment of reference values represents an unquestionable choice.

## Authors’ roles

A.P. conceived the study and analysed data; A.P., E.S. and P.V. wrote the manuscript; all the authors contributed to data analysis, critically discussed and approved the final version of the paper.

## Funding

The study received no specific funding.

## Conflict of interest

E.S. is Deputy Editor for *Human Reproduction Open* and reports grants from Ferring, grants and personal fees from Merck, grants and personal fees from Theramex, grants and personal fees from Gedeon-Richter, outside the submitted work. P.V. reports grants from Theramex and consultancy fees from Gedeon-Richter and Merck, outside the submitted work.

## Supplementary Material

deac161_Supplementary_Figure_S1

deac161_Supplementary_Table_SI
