# Supplementary material for: The statistical foundation of the reference population for semen analysis included in the sixth edition of the WHO manual: a critical reappraisal of the evidence
Source: Hum Reprod. 2022 Jul 18;37(10):2237–45. doi: 10.1093/humrep/deac161 (PMC9527466; doi:10.1093/humrep/deac161)
Supplement: deac161_Supplementary_Figure_S1 [file deac161_supplementary_figure_s1.pdf]

Human Reproduction, pp. 1–1, 2022
https://doi.org/10.1093/humrep/deac161

human reproduction

SUPPLEMENTARY DATA

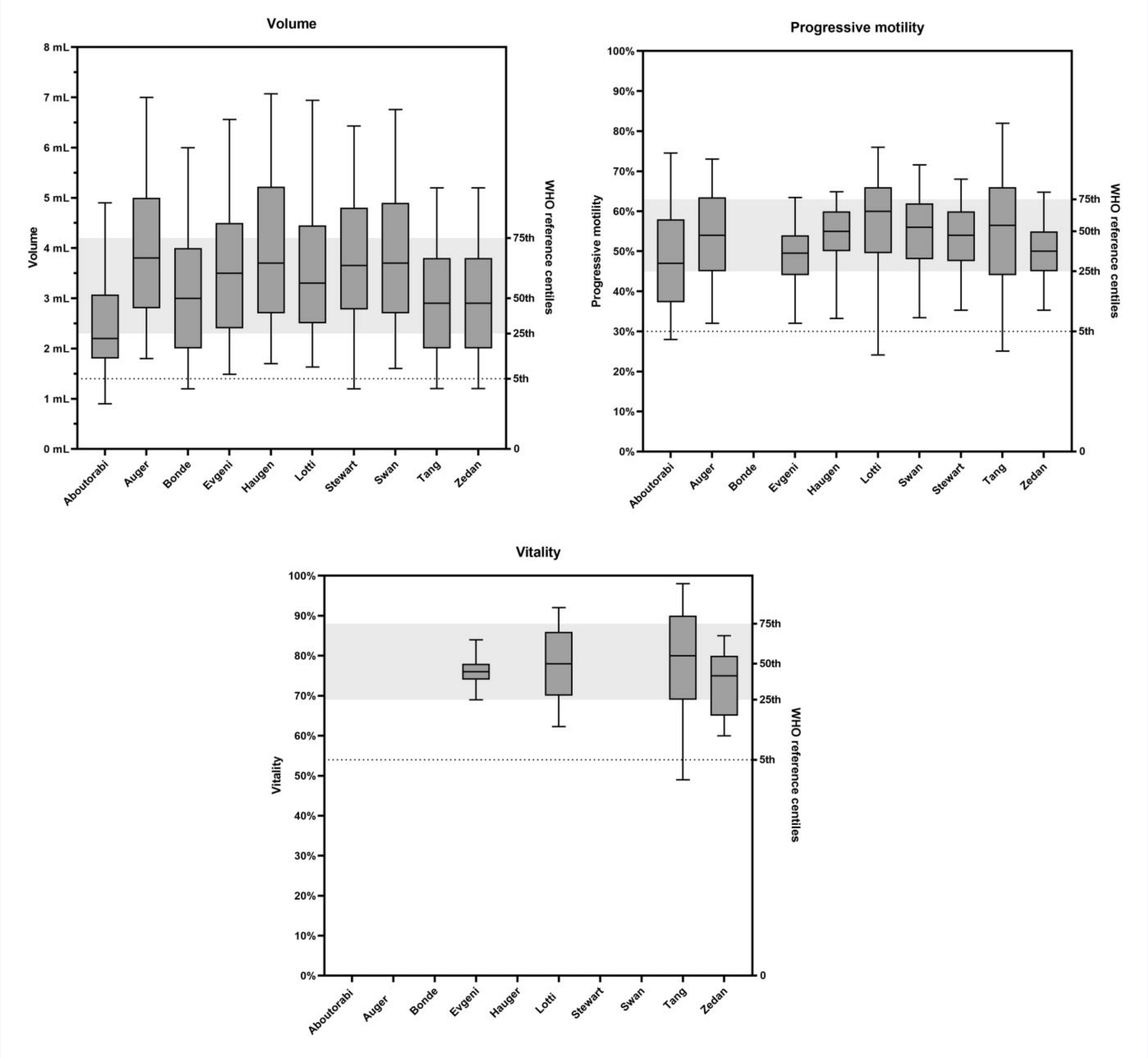

Supplementary Figure S1. Semen volume, progressive sperm motility and vitality in different studies. Boxes represent values between the 25th and 75th percentile; whiskers represent the 5th and 95th centiles. Ranges reported for the reference population in the 2021 World Health Organization (WHO) manual are indicated on the right *Y*-axes: the grey area represents the interquartile range, while the dotted line represents the 5th centile.
