# Supplementary material for: The statistical foundation of the reference population for semen analysis included in the sixth edition of the WHO manual: a critical reappraisal of the evidence
Source: Hum Reprod. 2022 Jul 18;37(10):2237–45. doi: 10.1093/humrep/deac161 (PMC9527466; doi:10.1093/humrep/deac161)
Supplement: deac161_Supplementary_Table_SI [file deac161_supplementary_table_si.pdf]

Human Reproduction, pp. 1–1, 2022
https://doi.org/10.1093/humrep/deac161

human reproduction

SUPPLEMENTARY DATA

Supplementary Table SI Comparison of reference values among different studies.

Study		Volume (ml)			Progressive motility (%)			Vitality (%)		
		5th	50th	75th	5th	50th	75th	5th	50th	75th
Aboutorabi *et al.* (2018)	*Reference value*	*1.5*	*3.2*	*4.3*	*30*	*55*	*63*	\	\	\
	Subjects in the range	17%**	80%**	91%**	7%	68%**	87%**			
Auger *et al.* (2001)	*Reference value*	*1.2*	*3.0*	*4.0*	*29*	*55*	*62*	\	\	\
	Subjects in the range	2%*	30%*	56%*	3%*	55%**	72%*			
Bonde *et al.* (1998)	*Reference value*	*1.4*	*3.1*	*4.3*	\	\	\	\	\	\
	Subjects in the range	8%**	59%**	82%**						
Evgeni *et al.* (2015)	*Reference value*	*1.4*	*3.0*	*4.3*	*30*	*55*	*63*	*54*	*78*	*88*
	Subjects in the range	4%	45%	72%	1%	82%**	96%**	0%*	82%**	100%**
Haugen *et al.* (2006)	*Reference value*	*1.4*	*3.0*	*4.2*	*30*	*55*	*63*	\	\	\
	Subjects in the range	2%	32%*	60%*	4%	56%	93%**			
Lotti *et al.* (2020)	*Reference value*	*1.4*	*3.0*	*4.2*	*30*	*54*	*62*	*53*	*78*	*88*
	Subjects in the range	3%	44%	74%	7%	37%*	61%*	0%*	53%	87%**
Stewart *et al.* (2009)	*Reference value*	*1.4*	*3.0*	*4.3*	*30*	*55*	*63*	\	\	\
	Subjects in the range	7%	37%*	69%*	3%	58%**	80%**			
Swan *et al.* (2003)	*Reference value*	*1.4*	*3.0*	*4.1*	*30*	*54*	*63*	\	\	\
	Subjects in the range	4%	33%*	64%*	3%	43%*	80%**			
Tang *et al.* (2015)	*Reference value*	*1.4*	*3.4*	*4.7*	*32*	*54*	*61*	*60*	*75*	*80*
	Subjects in the range	6%	70%**	91%**	10%**	46%	65%	13%**	48%	74%
Zedan *et al.* (2018)	*Reference value*	*1.4*	*3.1*	*4.3*	*29*	*55*	*63*	*53*	*79*	*89*
	Subjects in the range	5%	72%**	82%**	1%*	84%**	95%**	2%*	67%**	96%**

Reference values were calculated with the bootstrap non-parametric method excluding the study reported on the left. Subjects in the range are those described in the study reported on the left.
*The percentage of subjects with results in the specified percentile is significantly lower than expected.
**The percentage of subjects with results in the percentile is significantly higher than expected.
